# Supplementary figures and images for: Tailored lymph node dissection in right hemicolectomy: a retrospective study focusing on the anterior tissue of the superior mesenteric vein surgical trunk
Source: PeerJ. 2025 Apr 18;13:e19290. doi: 10.7717/peerj.19290 (PMC12011013; doi:10.7717/peerj.19290)

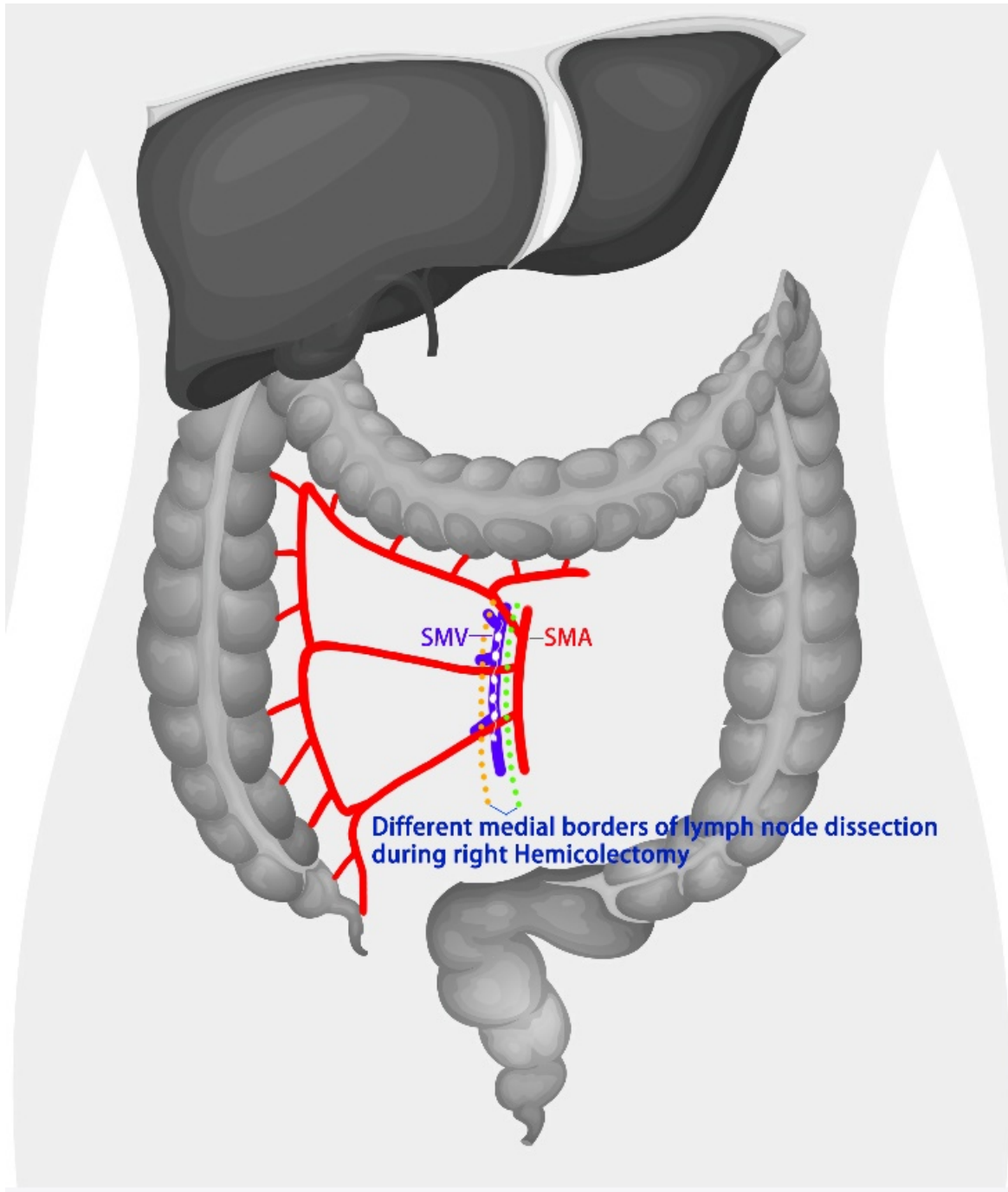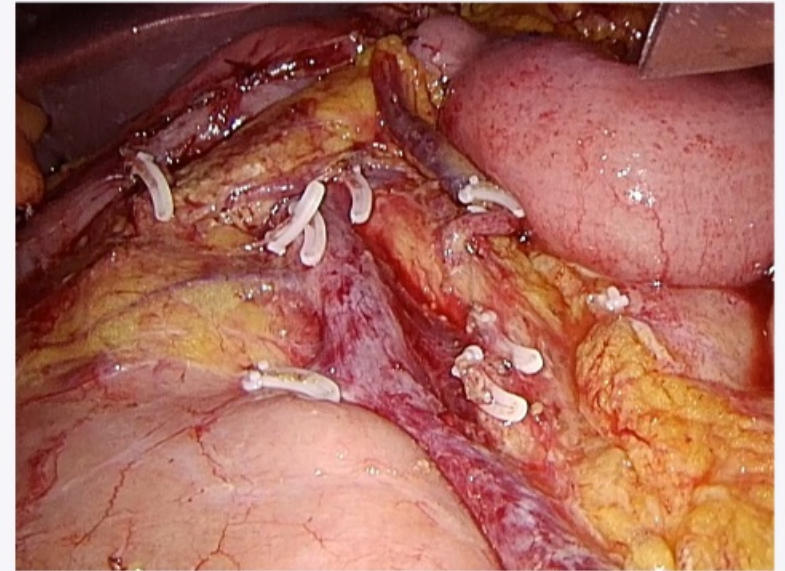

SMV-left

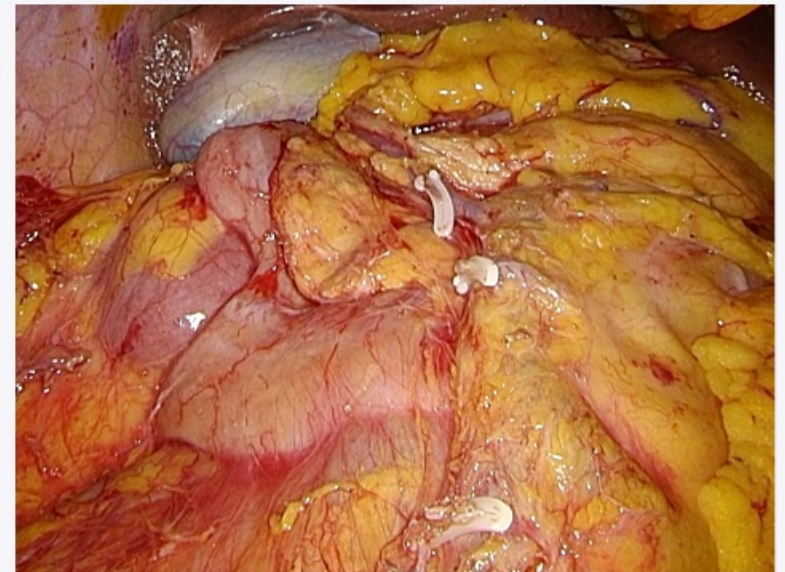

SMV-right

Supplement: Supplemental Information 2 — The SMV-left group involves dissection extending to the left side of the SMV and removal of the anterior tissue of the SMV surgical trunk. In contrast, the SMV-right group involves limited lymph node dissection to the right side of the SMV. SMA, Superior Mesenteric Artery. SMV, Superior Mesenteric Vein. [file peerj-13-19290-s002.pdf]
